# After insufficient radiofrequency ablation, tumor-associated endothelial cells exhibit enhanced angiogenesis and promote invasiveness of residual hepatocellular carcinoma

**DOI:** 10.1186/1479-5876-10-230

**Published:** 2012-11-21

**Authors:** Jian Kong, Lingqun Kong, Jinge Kong, Shan Ke, Jun Gao, Xuemei Ding, Lemin Zheng, Huichuan Sun, Wenbing Sun

**Affiliations:** 1 Department of Hepatobiliary Surgery, Beijing Chaoyang Hospital, Capital Medical University, Beijing, China; 2 Liver Cancer Institute, Zhongshan Hospital, Fudan University, Shanghai, China; 3 The Institute of Cardiovascular Sciences and Institute of Systems Biomedicine, School of Basic Medical Sciences, Peking University Health Science Center, Key Laboratory of Molecular Cardiovascular Sciences of Education Ministry, and Key Laboratory of Cardiovascular Molecular Biology and Regulatory Peptides of Health Ministry, Beijing, China

**Keywords:** Radiofrequency ablation, Hepatocellular carcinoma, Tumor-associated endothelial cells, Invasiveness, Metastasis

## Abstract

**Background:**

The mechanism regarding rapid progression of residual hepatocellular carcinoma (HCC) after insufficient radiofrequency ablation (RFA) has been preliminarily discussed. However, most studies have mainly focused on RFA-induced changes in the tumor cells. The present study was designed to determine whether tumor-associated endothelial cells (TAECs) could contribute to the invasiveness of HCC after insufficient RFA.

**Methods:**

TAECs were isolated from fresh HCC tissue and characterized. Morphological changes were observed in TAECs after heat treatment for 10 min. TAEC proliferation, migration and tube formation after heat treatment for 10 min at 37°C (control group), and 42 and 47°C (insufficient RFA groups) were examined. The differences in TAECs interactions with HepG2-GFP or HCCLM3-GFP cells among the two insufficient RFA groups and control group were evaluated. The expression of E-selectin, ICAM-1 and VCAM-1 in TAECs was measured. The effects of TAECs on the invasiveness of HepG2-GFP or HCCLM3-GFP cells after insufficient RFA were analyzed. The IL-6, IL-8, MCP-1 and GRO-α concentrations in conditioned medium from TAECs were measured after insufficient RFA. The associated signaling pathways of Akt, ERK1/2, STAT3 and NF-κB were analyzed in TAECs after insufficient RFA.

**Results:**

TAECs expressed the EC-specific markers and took up complexes of Dil-Ac-LDL. Relative to the control group, the proliferation of TAECs was significantly inhibited and their migration and tube formation were significantly enhanced in the insufficient RFA groups. Significantly more HepG2-GFP or HCCLM3-GFP cells adhered to TACEs in these groups than in the control group (all *P*<0.001), via up-regulated expression of E-selectin, ICAM-1 and VCAM-1. TAECs promoted the invasiveness of HepG2-GFP or HCCLM3-GFP cells after insufficient RFA via the up-regulation of IL-6, IL-8, MCP-1 and GRO-α in conditioned medium (all *P*<0.05). Insufficient RFA enhanced the activities of Akt, ERK1/2 and NF-κB signaling pathways and inhibited STAT3 signaling pathways.

**Conclusions:**

Insufficient RFA enhanced TAEC migration and tube formation, and this may play a key role in the rapid growth of residual HCC. Increased expression of metastasis-related molecules in TAECs after insufficient RFA may be a potential mechanism for the metastasis of residual HCC.

## Background

Hepatocellular carcinoma (HCC) is the sixth most common neoplasm and the third most frequent cause of cancer death [1]. Most HCC patients have underlying cirrhosis, which complicates the management of their cancer, especially in the case of early HCC in the setting of well-compensated cirrhosis [2]. Recently, radiofrequency ablation (RFA) has been considered as being potentially curative for early-stage HCC in patients with or without surgical prospects owing to its ease of use, safety and cost-effectiveness, and the fact that it is minimally invasive [3]. However, one of the major problems with RFA is the difficulty in achieving complete tumor destruction [4]. Because of the heat sink effect of blood vessels or the fact that the periphery of the tumor is distant from the center of ablation, the target temperature for ablation can not be easily achieved throughout the tumor; consequently, residual HCC can be present after RFA [5].

In the past decade, rapid tumor progression and sarcomatous changes after RFA have been reported in increasing numbers of clinical centers [6-8]. In particular, rapid tumor progression after RFA, which may mostly be associated with the progression of residual HCC, has been gaining increasing attention, and several underlying mechanisms for this phenomenon have been proposed. Our previous studies have demonstrated that insufficient RFA could facilitate rapid progression in residual hepatic VX2 carcinoma due to the induction of the over-expression of several molecular factors, and also promoted angiogenesis in residual HCC via hypoxia-inducible factor-1α (HIF-1α)/vascular endothelial growth factor-A (VEGFA) [9,10]. Another study also showed that insufficient RFA therapy may induce further malignant transformation of HCC [11]. However, these studies have mainly been focused on RFA-induced changes to the tumor cells. Whether or not non-tumor cells are also affected by insufficient RFA, and consequently contribute to progression of tumor cells, is still poorly understood.

In primary tumors, cancer cells are surrounded by a complex microenvironment comprised of numerous cells including endothelial cells present in the blood and lymphatic circulation, stromal fibroblasts and a variety of bone marrow-derived cells [12]. Evidence is accumulating that the altered microenvironment after RFA may enhance the outgrowth of residual tumor cells [4,13-15]. Tumor-associated endothelial cells (TAECs), as an important component of the microenvironment of tumor, play a key role in angiogenesis. This is crucial for the growth and invasion of solid tumors, as the vasculature provides metabolic support for tumor cells and serves as the gatekeeper for their escape and entry into the circulation [16]. What is more, evidence also shows that TAECs express tumor-specific endothelial markers and contain several regulated secretory organelles. These include Weibel-Palade bodies, the tissue plasminogen activator (tPA) organelle, and the type-2 chemokine-containing organelle, which are responsible for the secretion of tPA, cytokines IL-8 and IL-6, monocyte chemoattractant protein-1 (MCP-1), and growth-regulated oncogene-α (GRO-α) [17-19]. It has yet to be established if insufficient RFA enhances angiogenesis in TAECs. Whether or not TAECs promote the invasiveness of residual HCC via secreted cytokines and help residual tumor cells intravasate into the circulation after insufficient RFA also requires further investigation.

In the present study, we explored the biological behavioral changes in TAECs after insufficient RFA, and whether or not insufficient RFA promoted the metastasis of hepatoma cells via the enhanced secretion of cytokines and expression of adhesion molecules in TAECs.

## Materials and methods

### Patients

Fresh HCC tissues were obtained from seven patients who had positive α-fetoprotein (AFP) with curative liver resection at the Liver Cancer Institute and Zhongshan Hospital, Fudan University (Shanghai, China). The study design was approved by the Ethics Committee of Fudan University, and all the study participants provided informed consent.

### Cell lines and cell culture

Stable green fluorescent protein (GFP)-expressing HepG2 and HCCLM3 cells, two human HCC cell lines established at the Liver Cancer Institute and Zhongshan Hospital [20,21], were maintained in high-glucose DMEM (Gibco, Los Angeles, USA) supplemented with 10% fetal bovine serum (FBS; Gibco, Los Angeles, USA), 100 U/ml penicillin and 100 μg/ml streptomycin in a humidified atmosphere of 5% CO_2_ at 37°C. TAECs were isolated as described before [22]. Briefly, TAECs were obtained from surgical HCC specimens immediately after removal from patients. Specimens were minced and digested by incubation for 1 h at 37°C in 1640 medium (Gibco, Los Angeles, USA) containing 0.1% collagenase IV (Sigma-Aldrich, St. Louis, MO, USA). After washing in PBS (Gibco, Los Angeles, USA), the cell suspension was forced through a graded series of meshes to separate the cell components from stroma and aggregates. TAECs were isolated from cell suspension using anti-CD31 monoclonal antibodies (mAbs) coupled to magnetic beads (Miltenyi Biotech, Bergisch Gladbach, Germany) and magnetic cell-sorting using the MACS system (Miltenyi Biotech, Bergisch Gladbach, Germany). To increase the purity of isolated TAECs after positive magnetic bead isolation, the cell pellets underwent a second isolation with anti-CD31 mAbs. Cells were grown in complete EGM-2 medium (Lonza, Basel, Switzerland) supplemented with 10% FBS, 100 U/ml penicillin and 100 μg/ml streptomycin in a humidified atmosphere of 5% CO_2_ at 37°C. TAECs were used at passages 1–6.

### Immunofluorescence analysis

TAECs were grown on 24-well plates to 40-50% confluence, then fixed and blocked. Cells were then incubated with primary monoclonal or polyclonal antibodies against CD31, CD34, vascular endothelial growth factor receptor-1 (VEGFR1), vascular endothelial growth factor receptor-2 (VEGFR2), von Willebrand factor (vWF), CD68 and αSMA (Santa Cruz, California, USA) overnight at 4°C. The next day, plates were washed and incubated with anti-mouse or anti-rabbit fluorescein isothiocyanate- and/or tetramethyl rhodamine isothiocyanate-conjugated secondary antibody (Invitrogen, Carlsbad, USA). Cells were counterstained with 4'-6-diamidino-2-phenylindole (DAPI; KeyGen Biotech, Nanjing, China) to visualize cell nuclei and observed using an inverted fluorescence microscopy (Olympus IX51) equipped with an Olympus Qcolor 3 digital camera (Olympus).

### Internalization of acetylated low-density lipoprotein

TAECs were incubated in serum-free EBM-2 medium containing 10 μg/ml rhodamine-labeled 1,1V-dioctadecyl-3,3,3V,3V-tetramethylindocarbocyanine acetylated low-density lipoprotein (Dil-Ac-LDL; Sigma-Aldrich, St. Louis, MO, USA) for 4 h at 37°C. The cells were fixed in 4% paraformaldehyde for 20 min at room temperature. Cells were counterstained with DAPI to visualize cell nuclei and analyzed using an inverted fluorescence microscope (Olympus IX51) equipped with an Olympus Qcolor 3 digital camera (Olympus).

### Heat treatment

TAECs were seeded onto the 6-cm dishes, and after 24-h incubation they were exposed to heat treatment. Heat treatment was carried out by sealing the tops of culture plates with parafilm, and submerging the plates in a water bath set to the desired temperature for 10 min. We selected heat treatments of 42 and 47°C for 10 min to simulate the effects of insufficient RFA and 37°C for 10 min as the control treatment *in vitro*. Cell morphological changes were observed using an inverted fluorescence microscope (Olympus IX51) equipped with an Olympus Qcolor 3 digital camera (Olympus).

### TAEC proliferation, migration and tube formation after heat treatment

TAEC proliferation was measured using a Cell Counting Kit-8 (CCK-8; Dojindo, Kumamoto, Japan) according to the manufacturer's instructions. Briefly, TAECs were cultured in 96-well plates at a concentration of 3 × 10^3^/well. After 24-h incubation, the plates were heat treated for 10 min at 37, 42 or 47°C. After incubation for 24, 48 or 72 h, 5 μL of CCK-8 reagent was added to each well. The absorbance was measured at 450 nm after 2.5-h incubation at 37°C.

TAECs were plated into the 6-well plates, and after 24-h incubation the plates were sealed and submerged for 10 min in a water bath set to 37, 42 or 47°C. At 24, 48 or 72 h after heat treatment, TAECs were trypsinized and resuspended for further experiments involving migration and tube formation.

Quantitative cell migration assays were performed using a modified Boyden chamber (Costar-Corning, New York, USA) with 8.0-μm pore polycarbonate filter inserts in 24-well plates as described previously [9]. Briefly, the lower chamber was filled with EGM-2 with 10% FBS, and TAECs (5 × 10^4^ cells/well) in serum-free medium were added into the upper chamber. The cells were allowed to migrate for 5 h at 37°C. The non-migrated cells were removed from the upper surface of the membrane by scraping with a cotton swab, and the migrating cells were fixed with methanol, stained with crystal violet (Beyotime, Nantong, China) and photographed under an inverted fluorescence microscope (Olympus IX51) equipped with an Olympus Qcolor 3 digital camera (Olympus). Migration was assessed by counting the number of stained cells from 10 random fields at ×100 magnification.

TAECs tube formation was studied on growth factor-reduced Matrigel (Becton Dickinson, San Jose, USA) diluted 1:1 in ice with cold EBM-2 in a 96-well plate. Cells (1 × 10^4^ cells/well) were added to Matrigel-coated 96-well plates. TAECs were photographed under an inverted fluorescence microscope (Olympus IX51) equipped with an Olympus Qcolor 3 digital camera (Olympus). Tube formation was assessed by counting the number of loops from 10 random fields at ×50 magnification.

### Collection of the conditioned medium and cell protein

TAECs were cultured in a T25 culture bottle at a concentration of 5 × 10^5^ cells in EGM-2 containing 10% FBS. After 24-h incubation, 1 ml of fresh EBM-2 was added to the culture bottle with replacement of EGM-2. The culture bottles were submerged in a water bath for 10 min at 37, 42 or 47°C. Immediately after heat treatment, 2 ml of fresh EBM-2 was added to each culture bottle and cells were cultured in the incubator at 37°C. After 24-h incubation, the medium was collected and spun down at 3000 rpm for 20 min, and the supernatant was collected and stored at −80°C. Cells were lysed using cell lysis buffer (150 mM NaCl; 50 mM Tris–HCl; pH 8.0; 0.1% SDS; and 1% Triton X-100) containing protease, phosphatase inhibitors, and protein was used for further experiments.

### Matrigel invasion assay

The invasiveness of hepatoma cells was assessed by measuring their ability to invade through Matrigel-coated transwell inserts with 8.0-μm pores (Costar-Corning, New York, USA). HepG2-GFP or HCCLM3-GFP cells were trypsinized and resuspended with conditioned medium. 200 μl of cell suspension (2 × 10^4^ or 5 × 10^4^ cells) was added to each upper well, and the lower chamber was filled with DMEM with 10% FBS. Hepatoma cells were allowed to invade for 36 h at 37°C. The non-invaded cells were removed from the upper surface of the membrane by scraping with a cotton swab, and the invaded cells were fixed with methanol, stained with crystal violet, and photographed under inverted fluorescence microscopy (Olympus IX51) equipped with an Olympus Qcolor 3 digital camera (Olympus). Invasion was assessed by counting the number of stained cells from 10 random fields at ×100 magnification.

### Enzyme-linked immunosorbent assay

Cytokines secreted into the conditioned medium were quantified using an enzyme-linked immunosorbent assay (ELISA) kits (for IL-6, MCP-1, IL-8 and GRO-α; Boster, Wuhan, China) according to the manufacturer’s instructions. The concentration of cytokines was normalized to the total cellular protein.

### Western blot

Equivalent amounts of whole cell extracts were subjected to SDS-PAGE gel and transferred to nitrocellulose membranes. The membranes were blocked with 5% non-fat milk for 2 h and then incubated with respective primary antibody overnight at 4°C followed by the incubation with the appropriate HRP-conjugated secondary antibody for 1.5 h at room temperature. Blots were visualized with an ECL detection kit (Pierce, USA) and analyzed using Quantity One 1-D Analysis Software (Bio-Rad, Hercules, USA).

### TAEC-tumor cell adhesion assay

TAECs were plated onto 96-well plates and allowed to grow until complete confluence was achieved. The plates were submerged in a water bath for 10 min at 37, 42 or 47°C. After 24-, 48- or 72-h incubation, cells were rinsed with PBS twice, and HepG2-GFP or HCCLM3-GFP cells (1 × 10^4^ cells) in fresh DMEM were added to the wells. The plates were incubated at 37°C in 5% CO_2_ for 20 min. Non attached cells were removed by washing thrice with PBS and the attached hepatoma cells in each well were visualized under a microscope and counted using an inverted fluorescence microscope (Olympus IX51) equipped with an Olympus Qcolor 3 digital camera (Olympus).

### Cell surface adhesion molecules assay using cell ELISA

TAECs were plated onto 96-well plates and allowed to grow until 80% confluence. The plates were submerged in a water bath for 10 min at 37, 42 or 47°C. After 24-, 48- or 72-h incubation, cells were washed with PBS twice and fixed with PBS containing 4% paraformaldehyde at room temperature. The plates were blocked with 2% BSA at 37°C for 2 h. Cell surface expressions of adhesion molecules were determined by means of primary binding with specific antibody for VCAM-1, ICAM-1 or E-selectin (Santa Cruz, California, USA), followed by secondary binding with an HRP-conjugated goat anti-rabbit IgG antibody. Quantification was performed by determination of colorimetric conversion at OD at 450 nm of 3,3’,5,5’-tetramethylbenzidine using a TMB peroxidase EIA substrate kit (Bio-Rad, Hercules, USA).

### Statistical analysis

Student’s t test or the ANOVA test was used for comparison of two groups or three groups using GraphPad Prism (GraphPad Software Inc., La Jolla, CA). A P value of <0.05 was set as the level of statistical significance.

## Results

### Isolation and characterization of TAECs from tumor tissue

**Figure 1:**
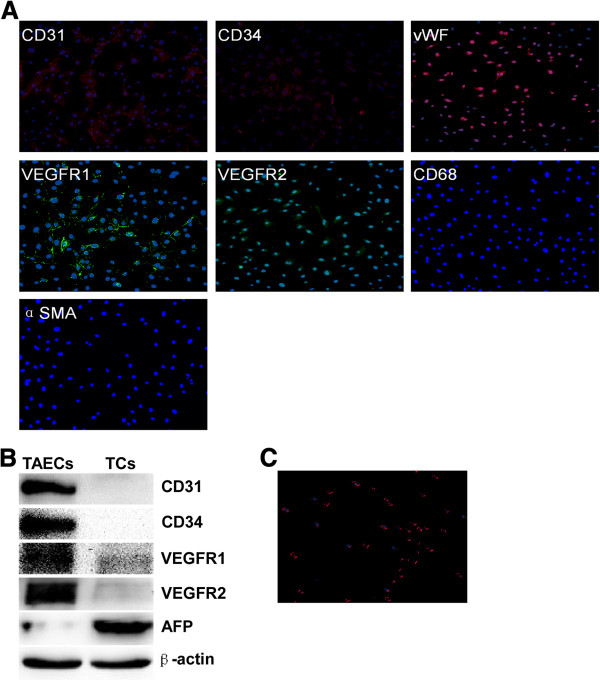
**Verification of TAECs from HCC.** (**A**) Representative immunofluorescence analysis of TAECs showing positive expression of the endothelial markers CD31, CD34, VEGFR1, VEGFR2 and vWF, and negative expression of the macrophages and fibroblast markers CD68 and α-SMA. (**B**) Western blot analysis used to verify the immunofluorescence results and assess the purity of the isolated CD31+ TAECs. (**C**) Micrograph representative of the uptake of Dil-Ac-LDL in TAECs. Five TAECs preparations were analyzed with similar results.

Seven strains of CD31+ TAECs were obtained from seven HCC patients. In addition to CD31, TAECs virtually expressed the EC-specific markers vWF, VEGFR1, VEGFR2 and CD34 (Figure 1A). Negative expression of CD68 and α-SMA in isolated TAECs excluded the contamination of macrophages and fibroblasts (Figure 1A). Western blot confirmed the immunofluorescence results and that AFP expression could be detected in tumor cells but not in TAECs (Figure 1B). TAECs also took up complexes of Dil-Ac-LDL (Figure 1C).

### The effect of heat treatment on TAECs

**Figure 2:**
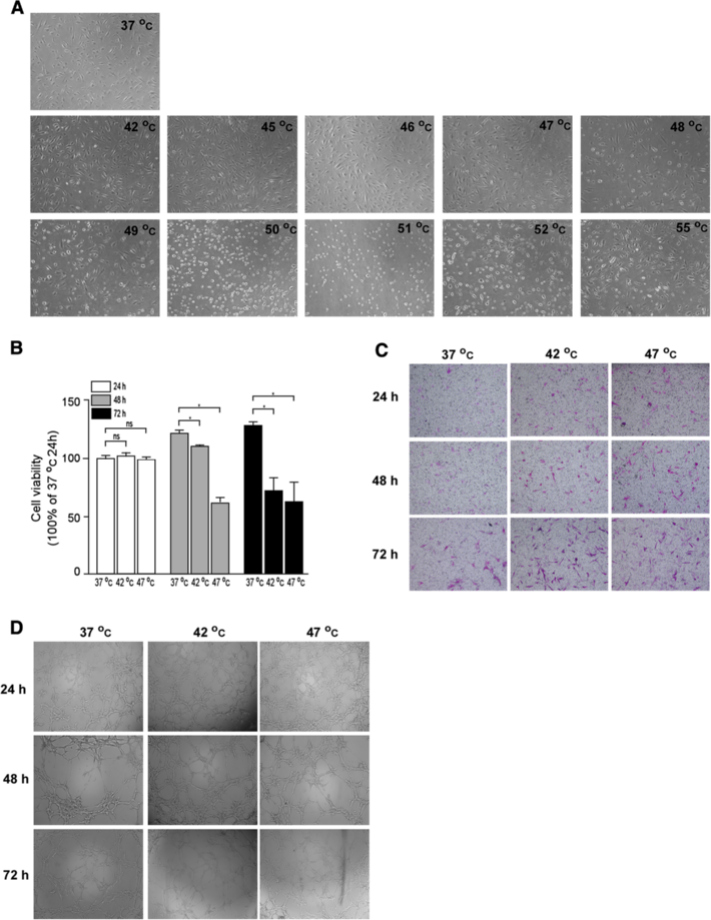
**Morphological changes in TAECs after heat treatment at different temperatures and inhibition of their proliferation and enhancement of their migration and tube formation after insufficient RFA.** (**A**) Representative micrograph of morphological change in TAECs after a 10-min heat treatment at temperatures ranging from 37 to 55°C. (**B**) TAECs were cultured after insufficient RFA (42 or 47°C treatment for 10 min). The proliferation of TAECs at 24, 48 and 72 h after heat treatment were measured using the CCK-8 assay. Columns: means from three individual experiments with five samples per group; bars: SE; *: *P* <0.05; ns: no significance. (**C**) TAECs were cultured after insufficient RFA and the migration of TAECs at 24, 48 and 72 h were measured using the trans-well assay. Representative micrographs of migration are shown. (**D**) TAECs were cultured after insufficient RFA and TAEC tube formation at 24, 48 and 72 h was evaluated using the Matrigel tube formation assay. Representative micrographs of tube formation are displayed. Data represent the representative results from three independent experiments with five samples per group.

In order to simulate the growth pattern of TAECs that were not killed after the heat stress generated by RFA, TAECs were exposed to a 10-min heat treatment at temperatures ranging from 37 to 55°C, and after 36 h, morphological changes in TAECs were observed. It was found that TAECs could not be continuously cultured once the temperature exceeded 47°C (Figure 2A). To monitor the potential effect of insufficient RFA on the function of TAECs *in vitro*, we initially observed the cell proliferation, migration and tube formation of TAECs at 24, 48 and 72 h after heat treatment. It was found that the proliferation of TAECs after insufficient RFA was significantly decreased as compared with the control treatment (Figure 2B). However, it was interesting that the insufficient RFA treatment promoted the migration and tube formation of TAECs; this was not observed after the control treatment (Figure 2C and D).

### Promotion of adhesion of TAECs to hepatoma cells after insufficient RFA

**Figure 3:**
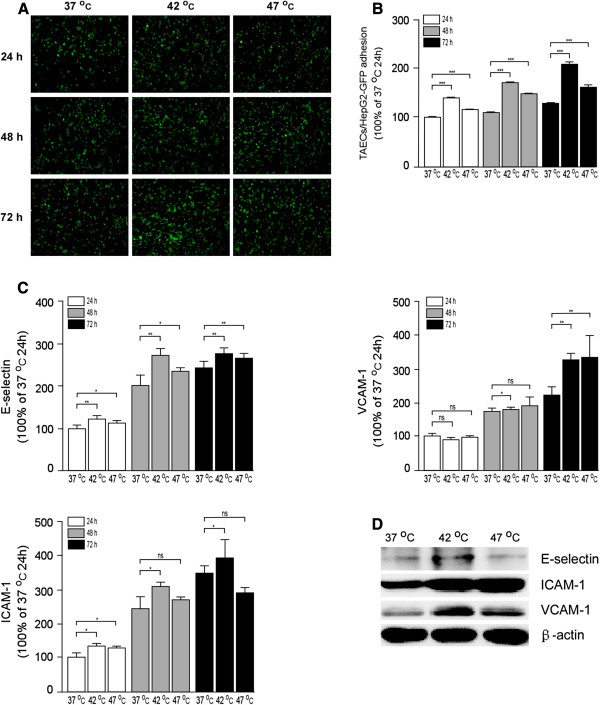
**Increased TAEC interaction with tumor cells and up-regulated expression of E-selectin, ICAM-1 and VCAM-1 after insufficient RFA.** (**A**-**B**) TAECs were cultured after insufficient RFA, and HepG2-GFP cells were added after 24, 48 and 72 h. Representative micrographs of TAECs regarding the interaction with HepG2-GFP cells are shown. Columns: means from three individual experiments with five samples per group; bars: SE; ***: *P* <0.001. (**C**) The expression of E-selectin, ICAM-1 and VCAM-1 on the surface of TAECs after insufficient RFA was detected using cell ELISA analysis. Columns: means from three individual experiments with five samples per group; bars: SE; *: *P* <0.05; **: *P* <0.01; ns: no significance. (**D**) Expression of E-selectin, ICAM-1 and VCAM-1 in TAECs after insufficient RFA detected by western blot analysis. Data are the representative results of three independent experiments with five samples per group.

HepG2-GFP or HCCLM3-GFP cells were used to investigate the adhesion ability of TAECs after insufficient RFA. TAECs were treated with insufficient RFA or the control treatment. After a 24-, 48- or 72-h interval, they were incubated at 37°C and hepatoma cells were added. The results showed that after insufficient RFA TACEs adhered to HepG2-GFP cells at a significantly higher level than the case after the control treatment (all *P* <0.001; Figure 3A and B). Similar results were observed in HCCLM3-GFP cells (all *P* <0.001; Additional file 1: Figure S1). In order to explore the mechanism involved in the process, we measured the surface expressions of the TAEC adhesion molecules after insufficient RFA using cell ELISA. The results showed that the expression of E-selectin, intercellular adhesion molecule (ICAM)-1 and vascular cell adhesion molecule (VCAM)-1 were significantly up-regulated on the surface of TAECs at 24, 48 and 72 h after insufficient RFA (Figure 3B). Western blot also confirmed the cell ELISA results (Figure 3C).

### Promotion of the invasiveness of hepatoma cells by TAECs after insufficient RFA

**Figure 4:**
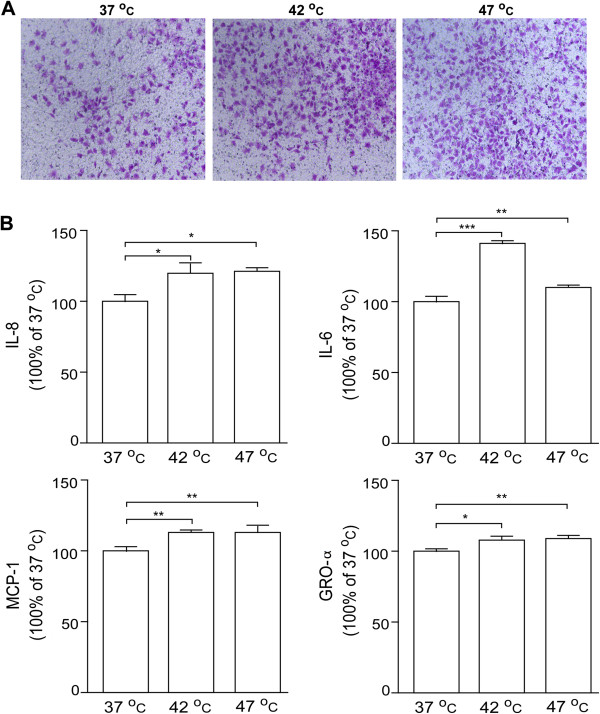
**Promotion of the invasiveness of hepatoma cells by TAECs after insufficient RFA.** (**A**) HepG2-GFP invasion *in vitro* in response to conditioned media from TAECs was assayed after the control treatment or insufficient RFA. Data are the representative results of three independent experiments with five samples per group. (**B**) IL-6, IL-8, MCP-1 and GRO-α concentrations in conditioned medium were detected using the ELISA assay. Columns: means from three individual experiments with five samples per group; bars: SE; *: *P* <0.05; **: *P* <0.01; ***: *P* <0.001.

Using the conditioned media from TAECs with or without insufficient RFA treatment, we further explored the effect of TAECs on the invasiveness of hepatoma cells. Conditioned medium from TAECs after insufficient RFA significantly enhanced the invasiveness of HepG2-GFP cells relative to the control (Figure 4A). Similar results were observed in HCCLM3-GFP cells (Additional file 2: Figure S2). To test the possible mechanism involved in the promotion of the invasiveness of hepatoma cells by TAECs after insufficient RFA, we measured the levels of cytokine secreted by TAECs in the conditioned medium. We found that insufficient RFA significantly increased the secreted levels of IL-8, IL-6, MCP-1 and GRO-α by TAECs (all *P*<0.05; Figure 4B).

### Enhancement of the activity of ERK1/2, NF-κB and Akt signaling pathways and inhibition of STAT3 signaling pathway after insufficient RFA

**Figure 5:**
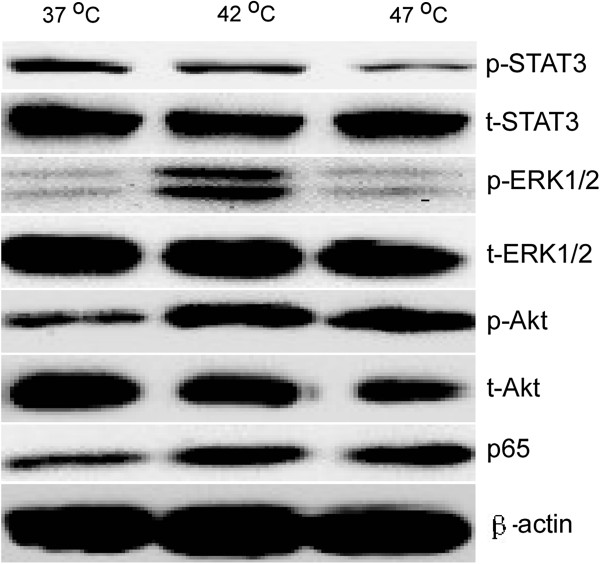
**Enhanced activity of ERK1/2, NF-κB and Akt signaling pathways and inhibition of the STAT3 signaling pathway after insufficient RFA.** The changes in signaling pathways involving TAECs after insufficient RFA were detected using western blot. Data are the representative results of three independent experiments with five samples per group.

To further determined the associated signal pathways involved in the process as described above, we investigated the expression levels of total and phosphorylated ERK1/2, NF-κB, Akt and STAT3 protein in TAECs at 24 h after insufficient RFA. It was found that total protein levels of ERK1/2, NF-κB, Akt and STAT3 were not changed after insufficient RFA, whereas phosphorylated ERK1/2 (p-ERK1/2), NF-κB and p-Akt were up-regulated and p-STAT3 was substantially down-regulated in TAECs after insufficient RFA (Figure 5).

## Discussion

RFA heats tumor tissue owing to ionic friction generated by the radiofrequency current, which induces coagulation necrosis once the tissue temperature exceeds 50°C for 4–6 min [23]. If the HCC tumor is not completely coagulated, the residual tumor cells are prone to proliferation, invasion and angiogenesis [9-11]. On the other hand non-tumor cells, especially TAECs, are also exposed to RFA, and insufficient RFA can theoretically influence the behavior of these cells. It remains poorly understood as to whether or not TAECs promote the metastasis of hepatoma cells after insufficient RFA.

The growth and migration of endothelial cells are essential for tumor angiogenesis [24]. In the absence of local neovascular formation, the tumor may not grow beyond 2–3 mm in diameter [25]. Most of the previous studies on tumor angiogenesis have been conducted using normal endothelial cells (NECs) such as human umbilical vein endothelial cells. The use of NECs does not reflect the real tumor microenvironment. In contrast TAECs, which express tumor-specific endothelial markers, are cytogenetically abnormal and genetically unstable [18,19]. TAECs also manifest an increased angiogenesis capability and drug resistance, and display resistance to interferon γ as compared with NECs under hypoxia [22,26]. Accordingly, the use of TAECs as a tool to research angiogenesis virtually reflects the microenvironment of tumor. Here, for the first time, we have observed the effect of insufficient RFA on TAECs *in vitro*. We isolated the CD31+ TAECs from fresh HCC tissue and found that insufficient RFA enhanced the migration and tube formation of these cells, but inhibited their proliferation. The results revealed that after insufficient RFA TAECs may enhance autochthonous angiogenesis to promote the rapid growth of residual HCC. The reason that the proliferation of TAECs was inhibited may be that insufficient RFA induced cell apoptosis and/or mitotic failure over a short time period.

Metastasis results from completion of a complex succession of cell-biological events-collectively termed the invasion-metastasis cascade. The process of cancer cell metastasis consists of: (1) local invasion through the surrounding extracellular matrix (ECM) and stromal cell layers; (2) intravasation into the lumina of blood vessels; (3) survival of the rigors of transport through the vasculature; (4) arrest at distant organ sites; (5) extravasation into the parenchyma of distant tissues; (6) initial survival in these foreign microenvironments in order to form micrometastases; and (7) reinitiation of proliferative programs at metastatic sites, thereby generating macroscopic, clinically detectable neoplastic growths [27]. Each of these processes involves rate-limiting steps that are influenced by non-malignant cells in the tumor microenvironment [12]. Previous studies have also shown that the altered microenvironment promotes the outgrowth and metastasis of residual tumor cells. However, it has not previously been reported that a non-malignant cell type *in vitro* can contribute adversely to the altered microenvironment after insufficient RFA.

It is known that the first step in metastasis is local invasion of hepatoma cells through the surrounding ECM. In present study, we found that TAECs significantly promoted hepatoma cells cell invasion through Matrigel *in vitro* after insufficient RFA. This suggested that cytokines secreted by TAECs in conditioned medium after insufficient RFA led to the increased invasiveness of the hepatoma cells. TAECs can secrete many cytokines, for example IL-8, IL-6, MCP-1 and GRO-α [17]. IL-8 plays an important role in inflammation, tumor-induced angiogenesis and tumor metastasis and IL-6 is involved in the proliferation, differentiation and metastasis of various malignant tumor cells [28-30]. MCP-1 secreted by hepatic myofibroblasts promotes the migration and invasion of human hepatoma cells, and MCP-1 suppresses the inhibition of tumor growth and metastasis in lung cancer and HCC [31-33]. High serum levels of IL-6, IL-8 and MCP-1 have been shown to be positively correlated with tumor development in cancer patients [34-36]. GRO-α plays an important role with regard to disease progression and metastasis formation and has also been demonstrated to be positively associated with tumor size, stage, invasion, lymph node metastasis and patient survival in colorectal cancers [37,38]. In the present study, we discovered that cytokines secreted by TAECs including IL-6, IL-8, MCP-1 and GRO-α were up-regulated after insufficient RFA, which may explain the enhanced invasiveness ability of hepatoma cells.

The second step in metastasis involves the intravasation of hepatoma cells into the lumens of blood vessels, and the interaction of hepatoma cells with TAECs plays an important role in the process. In our study we observed that after insufficient RFA TACEs adhered to significantly more hepatoma cells. Meanwhile E-selectin, ICAM-1 and VCAM-1, which are all expressed on vascular endothelial cells, have been found to be responsible for the formation of firm adhesion between the tumor and the endothelium [39-42]. Our results revealed that insufficient RFA up-regulated the expression of E-selectin, ICAM-1 and VCAM-1 in TAECs, which suggests that up-regulated adhesion molecules may be the mechanism responsible for the enhanced adhesion of TAECs to hepatoma cells.

The biological changes in TAECs after insufficient RFA must involve various cell signal pathways. Activation of Akt, NF-κB, STAT3 and ERK1/2 was found in HCC, and this was associated with tumor cell survival, proliferation, invasiveness and metastasis [43-46]. In our previous study we also found that insufficient RFA activated the p-Akt/HIF-1α/VEGFA signal pathway of hepatoma cells promoting angiogenesis in residual HCC [9]. Our present study revealed that insufficient RFA could up-regulate the expression of p-ERK1/2, p-Akt and NF-κB and down-regulate the expression of p-STAT3 in TAECs. The up-regulated expression of p-ERK1/2, p-Akt and NF-κB may explain the phenomenon as described above. However, in the present study, we could not identify which signal pathway played a key role in the promotion of rapid growth and metastasis in residual HCC by TAECs, and why the expression of p-STAT3 in TAECs was down-regulated after insufficient RFA. Further study is needed in the future to clarify the exact mechanism involved in the signal pathway associated with the biological behavior of TAECs after insufficient RFA.

Combined with the findings from our previous research, our present results suggest that insufficient RFA affects not only hepatoma cells but also TAECs within the HCC. Such effects should be taken into account in the treatment of HCC using RFA. Anti-angiogenesis drugs, which are used to target TAECs, may be useful in preventing the rapid growth and metastasis of residual HCC after insufficient RFA.

## Conclusions

Enhanced TAEC migration and tube formation after insufficient RFA may play a key role in the rapid growth of residual HCC. Increased expression of metastasis-related molecules in TAECs after insufficient RFA may be a possible mechanism for the metastasis of residual HCC.

## Competing interests

The authors declare that they have no competing interests.

## Authors’ contributions

JK carried out the molecular biology studies, participated in the sequence alignment and drafted the manuscript. LQK and SK carried out the immunoassays. JGK, JG, LMZ and XMD participated in the sequence alignment. HCS participated in the design of the study and performed the statistical analysis. WBS conceived of the study, and participated in its design and coordination and helped to draft the manuscript. All authors read and approved the final manuscript.

## Supplementary Material

Additional file 1 **Figure S1.** Increased TAEC interaction with HCCLM3-GFP cells after insufficient RFA. (A-B) TAECs were cultured after insufficient RFA, and HCCLM3-GFP cells were added after 24, 48 and 72 h. Representative micrographs of TAECs regarding the interaction with HCCLM3-GFP cells are shown. Columns: means from three individual experiments with five samples per group; bars: SE; ***: *P* <0.001. Click here for file

Additional file 2 **Figure S2.** Promotion of the invasiveness of HCCLM3-GFP cells by TAECs after insufficient RFA. HCCLM3-GFP invasion *in vitro* in response to conditioned media from TAECs was assayed after the control treatment or insufficient RFA. Representative micrographs of HCCLM3-GFP cell invasion are shown. Data are the representative results of three independent experiments with five samples per group. Click here for file
