# Supplementary figures and images for: After insufficient radiofrequency ablation, tumor-associated endothelial cells exhibit enhanced angiogenesis and promote invasiveness of residual hepatocellular carcinoma
Source: J Transl Med. 2012 Nov 21;10:230. doi: 10.1186/1479-5876-10-230 (PMC3543343; doi:10.1186/1479-5876-10-230)

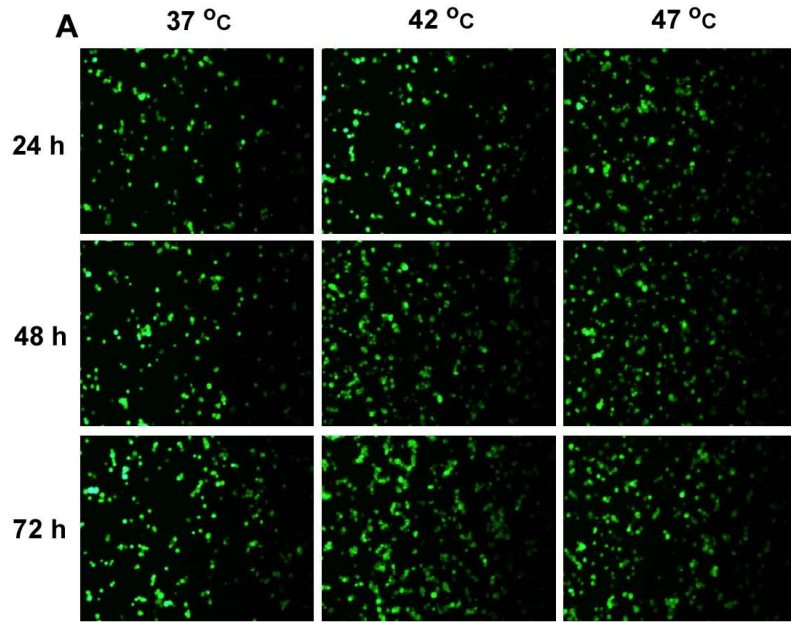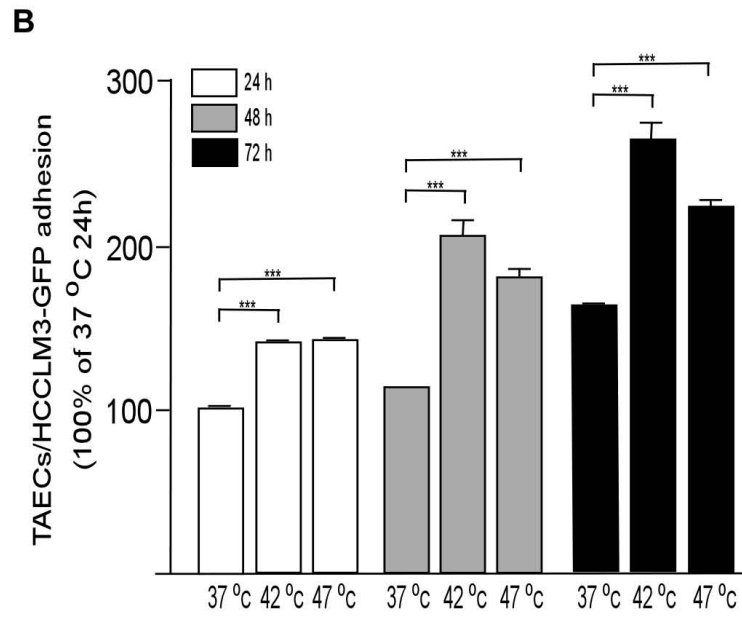

Supplement: Additional file 1 — Figure S1. Increased TAEC interaction with HCCLM3-GFP cells after insufficient RFA. (A-B) TAECs were cultured after insufficient RFA, and HCCLM3-GFP cells were added after 24, 48 and 72 h. Representative micrographs of TAECs regarding the interaction with HCCLM3-GFP cells are shown. Columns: means from three individual experiments with five samples per group; bars: SE; ***: P <0.001. [file 1479-5876-10-230-S1.pdf]

37 °C

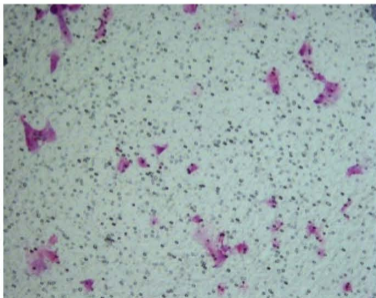

42 °C

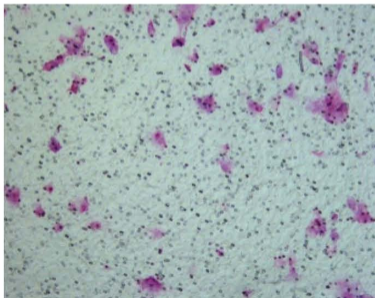

47 °C

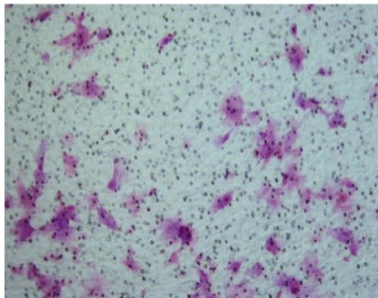

Supplement: Additional file 2 — Figure S2. Promotion of the invasiveness of HCCLM3-GFP cells by TAECs after insufficient RFA. HCCLM3-GFP invasion in vitro in response to conditioned media from TAECs was assayed after the control treatment or insufficient RFA. Representative micrographs of HCCLM3-GFP cell invasion are shown. Data are the representative results of three independent experiments with five samples per group. [file 1479-5876-10-230-S2.pdf]
